# Importance of understanding the main metabolic regulation in response to the specific pathway mutation for metabolic engineering of *Escherichia coli*

**DOI:** 10.5936/csbj.201210018

**Published:** 2013-01-16

**Authors:** Yu Matsuoka, Kazuyuki Shimizu

**Affiliations:** a Department of Bioscience and Bioinformatics, Kyushu Institute of Technology, Iizuka, Fukuoka 820-8502, Japan; b Institute of Advanced Bioscience, Keio University, Tsuruoka, Yamagata 997-0017, Japan

## Abstract

Recent metabolic engineering practice was briefly reviewed in particular for the useful metabolite production such as natural products and biofuel productions. With the emphasis on systems biology approach, the metabolic regulation of the main metabolic pathways in *E. coli* was discussed from the points of view of enzyme level (allosteric and phosphorylation/ dephosphorylation) regulation, and gene level (transcriptional) regulation. Then the effects of the specific pathway gene knockout such as *pts*, *pgi*, *zwf*, *gnd*, *pyk*, *ppc*, *pckA*, *lpdA*, *pfl* gene knockout on the metabolism in *E. coli* were overviewed from the systems biology point of view with possible application for strain improvement point.

## 1. Introduction

The concept of metabolic engineering has been proposed back in 1991 (Bailey, 1991; Stephanopoulos and Vallino, 1991). The main objective of metabolic engineering is to manipulate and modify the metabolism through the use of recombinant DNA technology for the improvement of the useful metabolite(s) production with typically the increase in yields, titers, and productivities. The objective has been further expanded to the enhancement of the substrate consumption rate in particular for the simultaneous consumption of multiple carbon sources obtained from lignocelluloses and waste materials, and the augmentation of cellular robustness such as the improved tolerance towards toxic compounds and acidic condition (Alper et al., 2006; Atsumi et al., 2010; Dunlop et al., 2011).

For metabolic engineering, following factors must be taken into account: (1) available raw materials such as cellulosic biomass, algae, and waste materials, which can be converted to glucose, xylose, fructose, galactose, mannitol, and glycerol etc. by the appropriate saccharification methods and their processing costs; (2) enhancement of the substrate uptake rate and co-consumption of multiple carbon sources (for the enhancement of the target metabolite production rate); (3) appropriate host microbes such as *Escherichia coli*, *Saccharomyces cerevisiae*, *Corynebacterium glutamicum*, *Bacillus subtilis* etc.; (4) metabolic pathways available in the strains from the available carbon source(s) to the target metabolite(s), (5) introduction of the heterologous pathways by recombinant DNA technology; (6) reduction of the flux to undesirable by-products; (7) enhancement of precursor metabolites production together with necessary cofactors production; (8) modulation of transporters for exporting the target metabolite(s) to the extracellular medium; (9) understanding the metabolic regulation mechanism by both allosteric regulation of enzymes and transcriptional regulation by global regulators; (10) robust and responsive genetic control system for the desired pathways and the host microbe, (11) methods for debottlenecking the chosen pathways; and (12) ways to maximize yields, titers, and productivities (Keasling, 2010; Yadav et al., 2012).

In industry, a limited number of platform cell factories is applied for the production of a wide range of fuels and chemicals (Nielsen and Keasling, 2011). The most popular platform cell factories may be *E. coli* by probably considering the advantages of high cell growth rate and well-known characteristics etc. In the field of biofuel production, much interest has centered on *S. cerevisiae*. *Corynebacterium glutamicum* is also frequently employed as platform cell factories in industry in particular for amino acid production. An overview of the recent advances in engineering the central carbon metabolism of such industrially important bacteria as *E. coli*, *C. glutamicum*, *B. subtilis*, *Streptomyces* sp., *Lactococcus lactis* has been reported (Papagianni, 2012).

One of the successful example for metabolic engineering practice in industry may be the production of natural products, particularly active pharmaceutical ingredients, some of which are too complex to be chemically synthesized and yet a value that justifies the cost for developing a genetically engineered microorganism (Keasling, 2010). One such example is alkaloids found primarily in and derived from plants and widely used as drugs (Hawkins and Smolke, 2008). Other examples are polyketides and nonribosomal peptides, which are used as veterinary agents and agrochemicals etc. Some of the most valuable molecules have been produced with engineered industrial host organisms (Pfeifer et al., 2001). Isoprenoids can be used as fragrances and essential oils, nutraceuticals, and pharmaceuticals, and many isoprenoids such as carotenoids and terpenes have been produced by microorganisms (Martin et al., 2003; Ro et al., 2006; Leonard et al., 2010; Chang et al., 2007).

In contrast to the above chemicals, solvents and polymer precursors are rarely produced by microorganisms in industry due to higher cost as compared to their production from inexpensive petroleum by chemical catalysis. However, petroleum prices are fluctuating with unstable political situations, and increasing trends due to limited source of petroleum and global warming pressure. The current trends for producing such inexpensive chemicals are to utilize low-cost resources such as lignocellulosic biomass, or starch etc. derived from agricultural and forest waste etc. One example is 1,3-propandiol, a useful intermediate in the synthesis of polyurethanes and polyesters, produced by recombinant *E. coli* (Nakamura and Whited, 2003). Another more popular examples are the production of transportation fuels such as ethanol and butanol by recombinant microorganisms (Munjal et al., 2012; Geddes et al., 2011; Edwards et al., 2011; Fontman et al., 2008; Atsumi et al., 2008a; Inui et al., 2008; Steen et al., 2008). Large branched-chain alcohols can be also produced by engineered microorganism (Atsumi et al., 2008b), where those may be considered to be better fuels than ethanol and butanol, and can be also used to produce a commodity chemicals. Metabolic engineering can be also applied to produce hydrocarbons with properties similar to those petroleum-derived fuels, where linear hydrocarbons such as alkanes, alkenes and esters, typical of diesel and jet fuel have been produced by way of fatty acid synthetic pathway (Steen et al, 2010; Beller et al., 2010; Schirmer et al., 2010). An overview on the recent development of microbial cell factories for bio-refinery through synthetic bioengineering has been reported (Kondo et al., 2012).

As stated above, the pathway engineering has been extensively applied so far. However, the performance improvement is somewhat limited. This may be due to the poor understanding on the overall metabolic regulation mechanism. Since it is quite important to properly understand the metabolic regulation mechanism in particular for the main metabolism, here we consider this based on systems biology approach with particular attention to the effect of the specific pathway gene mutation on the metabolism.

## 2. Systems biology approach

The important approach for metabolic engineering is the systems biology approach, where large genome-scale models have been developed (Palsson, 2009; Feist and Palsson, 2008; Herrgard et al., 2008), and the catabolite regulation mechanism in *E. coli* was modeled to some extent (Kotte et al., 2010; Bettenbrock et al., 2006; Kremling et al., 2001, 2004). Functional genomics are important for characterizing the molecular constituents of a cell system, where systems biology addresses the missing links between molecules and physiology (Westerhoff et al., 2009; Bruggeman and Westerhoff, 2006). In this approach, the integration of different levels of “omics” data is important. The perhaps most comprehensive data set containing transcriptomics, proteomics, metabolomics, and fluxes for *E. coli* grown at different growth rates in glucose-limited continuous cultures and upon deletion of 24 glycolysis and pentose phosphate pathway genes (Ishii et al., 2007) may provide an unprecedented opportunity for computational systems biology analyses to extract biological insight (Zamboni et al., 2007).

Classical metabolic control analysis (MCA) (Fell, 1997; Heinrich and Schuster, 1996) may be applied to identify the limiting pathway, or it can be extended to the regulation analysis in the case of nutrient starvation for *S. cerevisiae* (Rossell et al., 2005; Rossell et al., 2006). However, the success of MCA analysis highly relies on the accuracy of the model used.

Burgard et al. (2003) proposed the so-called OptKnock algorithm that implements bilevel programming for the optimization of both biomass and the specified metabolite production. This was extended for the case of gene amplification etc. (Pharkya et al., 2004; Pharkya and Maranas, 2006). Lee et al. (2005) considered *in silico* gene knockout simulations for succinic acid fermentation. Trinh et al. (2006) applied the concept of inverse metabolic engineering (Bailey et al., 1996) based on elementary mode analysis for maximizing the biomass yield of *E. coli*. Combinatory pathway analysis has also been used for overproduction of lycopene (Alper et al., 2005a), and for tyrosine production (Lutke-Eversloh and Stephanopoulos, 2008). Yet another approach to engineering the global cellular transcription machinery is to make global perturbations of the transcriptome, which can help to unravel complex phenotypes, and which has some applications to ethanol tolerance, and the specific metabolite overproduction and so on (Alper et al., 2007). Moreover, Alper et al. (2005b) considered a new framework for the quantitative control of gene expression through engineering promoters.

## 3. Importance of modulating the main metabolism

The cell's metabolism comprises thousands of reactions that are involved in the degradation of available nutrient sources for biosynthesis of cellular constituents such as proteins, lipids, carbohydrates, DNA and RNA etc. Note that those are formed from several key building blocks such as amino acids for protein synthesis, fatty acids for lipids synthesis, nucleotide for DNA and RNA synthesis, and sugar moieties for carbohydrate synthesis. Moreover, it should be noted that those building brocks are formed from only limited number of precursor metabolites generated in the main metabolism (Nielsen, 2003). These characteristics are common to any living organisms. Namely, such biosynthesis nature is conserved among all living organisms, where the 12 precursor metabolites such as G6P, F6P, R5P, E4P, GAP, 3PG, PEP, PYR, OAA, AcCoA, αKG, and SucCoA are formed in the central carbon metabolism from the available carbon sources (Varma and Palsson, 1993). For the cell metabolism to function, and for the cell to grow, several cofactors such as ATP, NADH, NADPH as well as other nutrient sources such as nitrogen, phosphate, metal ion etc. are requited. Since the central carbon metabolism is tightly connected with overall cell function, it is quite important to understand its regulation mechanism for metabolic engineering (Nielsen, 2011).

**Figure 1:**
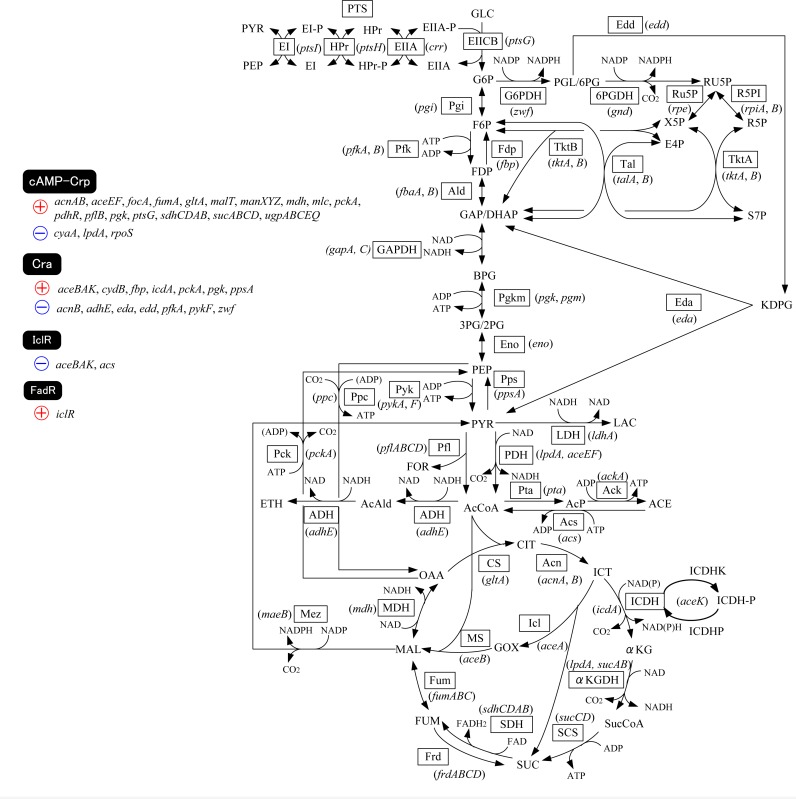
Main metabolic pathways of *E.coli* together with transcription factors.

**Figure 2:**
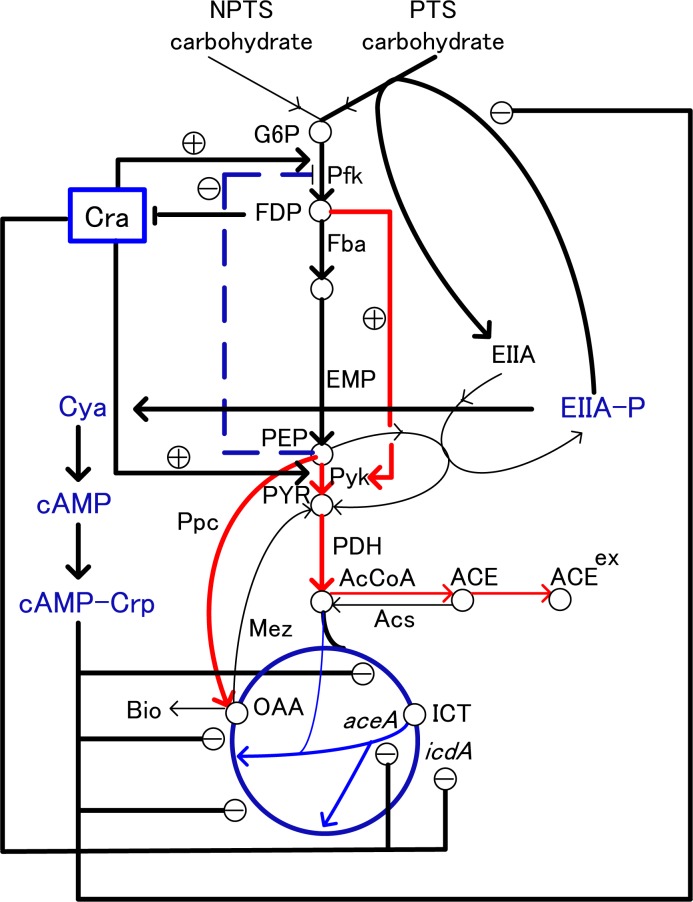
Overall regulation mechanism for the main metabolism by enzyme level regulation and transcriptional regulation.

Figure 1 shows the main metabolic pathways of *E. coli* together with transcription factors (Matsuoka and Shimizu, 2011). Suppose that the glucose uptake rate was increased, or PTS was enhanced, then glycolysis flux increases, and thus F16BP (FDP) concentration increases. The increased FDP allosterically enhances the activity of Pyk (and also Ppc) by feed-forward control (Figure 2). The PEP concentration tends to be decreased due to activation of Pyk (and Ppc), although it is not obvious since the glycolysis flux increases, and tends to give more PEP. It has been known that PEP molecule allosterically inhibits Pfk activity by feed-back regulation. The decrease in PEP concentration thus causes Pfk activity to be increased, and glycolysis flux and thus FDP concentration are more increased. On the other hand, the decrease in PEP and increase in PYR make PEP/PYR ratio to be decreased. This causes phosphorylated EIIA (EIIA-P) concentration to be decreased, and in turn less activates Cya (adenylate cyclase), and thus cAMP is less formed from ATP. As a result, cAMP-Crp level decreases, which decreases the expression of *ptsG* (Table 1), which codes for EIIBC, and this causes the decrease in glucose uptake rate. This forms a negative feed-back loop for the initial increase in glucose uptake rate (Figure 2). This indicates that PTS plays an essential role from the robustness point of view in addition to phosphorylation of substrate. Conversely, this robustness is not guaranteed for the case of non-PTS carbohydrate, *pts* mutant, and the cells without having PTS such as yeast etc.

**Table 1:** Regulation of global regulators on the metabolic pathway gene

Global regulator	Metabolic pathway gene
Cra	+: *aceBAK*, *cydB*, *fbp*, *icdA*, *pckA*, *pgk*, *ppsA*
	−: *acnB*, *adhE*, *eda*, *edd*, *pfkA*, *pykF*, *zwf*
Crp	+: *acnAB*, *aceEF*, *focA*, *fumA*, *gltA*, *malT*, *manXYZ*, *mdh*, *mlc*, *pckA*, *pdhR*, *pflB*, *pgk*, *ptsG*, *sdhCDAB*, *sucABCD*, *ugpABCEQ*
	−: *cyaA*, *lpdA*, *rpoS*
ArcA/B	+: *cydAB*, *focA*, *pflB*
	−: *aceBAK*, *aceEF*, *acnAB*, *cyoABCDE*, *fumAC*, *gltA*, *icdA*, *lpdA*, *mdh*, *nuoABCDEFGHIJKLMN*, *pdhR*, *sdhCDAB*, *sodA*, *sucABCD*
IclR	+: *aceBAK*, *acs*
FadR	−: *iclR*
Mlc	+: *crr*, *manXYZ*, *malT*, *ptsG*, *ptsHI*
PdhR	−: *aceEF*, *lpdA*
Fnr	+: *acs, focA, frdABCD, pflB, yfiD*
	−: *acnA, cyoABCDE, cydAB, fumA, fnr, icdA, ndh, nuoA-N, sdhCDAB, sucABCD*
RpoS	+: *acnA, acs, adhE, fumC, gadAB, talA, tktB, poxB, asmC*
	−: *ompF*

**Cra**: Carbon flow controller which activates gluconeogenetic pathway genes and repress glycolysis genes

**Crp**: Major global regulator for catabolite-sensitive operons (when complexed with cAMP)

**ArcA/B**: Regulator of the growth under micro-aerobic condition, where ArcB is a sensor protein.

**IclR**: Repressor of glyoxylate pathway genes

**FadR**: Regulator of fatty acid metabolism

**Mlc**: Repressor of the genes involved in carbohydrate utilization

**PdhR**: Regulator which controls the genes involved in PDHc

**Fnr**: Regulator for the growth under anaerobic (anoxic) condition

**RpoS**: Master stress regulator and σ^38^ or σ^S^, major sigma factor during stationary phase

The analysis may be further extended to the regulation of TCA cycle. As mentioned above, the increase in glucose uptake rate causes PYR concentration to be increased. Note that the increase in Ppc caused by the increase in FDP affects OAA concentration to be increased. Moreover, the increase in PYR concentration causes AcCoA to be increased, and thus acetate formation is enhanced by Pta-Ack reaction. The increase in AcCoA also enhances Ppc activity (Yang et al., 2003), where AcCoA is the activator of Ppc. The increases in AcCoA and OAA cause TCA cycle to be activated. On the other hand, as mentioned above, if PTS was furnished, cAMP-Crp decreases as glucose uptake rate increases, which in turn represses the expressions of TCA cycle genes such as *acnA*, *B*, *sucABCD*, *sdhCDAB*, *fumA*, and *mdh* (Table 1), and thus TCA cycle activity is repressed by transcriptional regulation (Figure 2). In fact, there is another global regulator Cra (FruR) that plays an important role for the control of carbon flows, where Cra detects FDP concentration, and Cra activity decreases with the increase of FDP concentration. In the above example, the increase in glucose uptake rate increases FDP concentration, and thus Cra activity decreases. This causes the increases of the expressions of glycolysis genes such as *pfkA* and *pykF* genes, while it represses the expressions of gluconeogenetic pathway genes such as *ppsA* and *pckA* genes (Table 1), which implies acceralation of increased glycolysis fluxes. The decrease in Cra activity also affects TCA cycle genes such that *icdA* and *aceA* gene expressions are repressed (Table 1), and thus TCA cycle is further repressed by this mechanism (Figure 2). The increase in glycolysis activity and the decrease in TCA cycle activity cause more acetate production.

## 4. Effect of the specific pathway mutation on the metabolic regulation of the main metabolism

The proper understanding of the metabolic regulation of the central carbon metabolism is critical for the efficient metabolic engineering. For this, it may be useful to understand the effect of the specific pathway mutation on the metabolic changes based on ^13^C-metabolic flux distribution together with different levels of information (Shimizu, 2004, 2009, 2013). Table 2 shows the summary of some of the past investigations on the metabolic changes caused by the specific gene(s) knockout in the main metabolic pathways in *E. coli*. While the specific-gene knockout mutations in the central metabolism preclude growth on glucose, a majority of such mutations seem to be potentially compensated by the use of alternative enzymes or by rerouting of the carbon fluxes through alternative pathways, resulting in a robust phenotype such as little effect on the cell growth rate.

**Table 2:** Effect of the specific gene mutation on the metabolism

Mutants	Main feature	Source
	NADPH overproduction with depressed cell growth	2003
Δ*pgi*	Anaprelotic pathway through Ppc decreases, while glyoxylate pathway is activated	Toya et al., 2010
		Usui et al., 2012
	Accumulation of PEP, which causes activation of Ppc and Mez pathways to backup PYR	Siddiquee et al., 2004
Δ*pykFA*		2007
	Increase in PEP and the activation of PP pathway (E4P), which activates the pathways toward aromatic amino acids	Toya et al., 2010
		Escalante et al., 2010
		Meza et al., 2012
Δ*ppc*	Decrease of OAA, which activates Glyoxylate pathway	2004
Δ*pckA*	Decrease of OAA, which activates Glyoxylate pathway	2003
Δ*ppsA*	Significant delay in the diauxic transition from glucose to acetate	2005
Δ*lpdA*	PYR is accumulated, and Pox and ACS as well as glyoxylate pathway are activated	2006a
Δ*pflA*,*B*	D-lactate overproduction under anaerobic condition	Zhu et al., 2004, 2005
Δ*ldhA*	Formate, acetate, and ethanol are more formed than wild type under anaerobic condition	2005
Δ*icdA*	Activates glyoxylate pathway and Mez	Kabir et al., 2006
Δ*sucA*,C	Glyoxylate pathway is activated in Δ*sucA* but not in Δ*sucC*	2006b
Δ*zwf*	Glycolysis is activated with acetate overflow metabolism PP pathway metabolites are given from F6P and GAP	2004a
	Mez is activated for NADPH formation	2003
Δ*gnd*	Oxidative PP pathway is repressed, and ED pathway is activated	2004b

Noting that the PTS prevents the expression of catabolic genes and the activity of non-PTS sugars transport system by carbon catabolite repression (CCR) for the case of using a mixture of multiple carbohydrates, an early attempt was to knockout *pts* gene. In the case of *E. coli*, the glucose molecule in the periplasm can be internalized into cytoplasm either by PTS, galactose permease (GalP) system and the MglBAC system (Gosset, 2005). Thus GalP and MglBAC together with glucokinase (Glk) play important roles for PTS^-^ mutants. However, those systems are less efficient from the energetic point of view, and as a result, the glucose consumption rate becomes significantly low. In order to overcome this problem and attain co-consumption of PTS and non-PTS sugars, *galP* and *glk* genes were implemented (Flores et al., 2007; Escalante et al., 2012; Lu et al., 2012).

**Figure 3:**
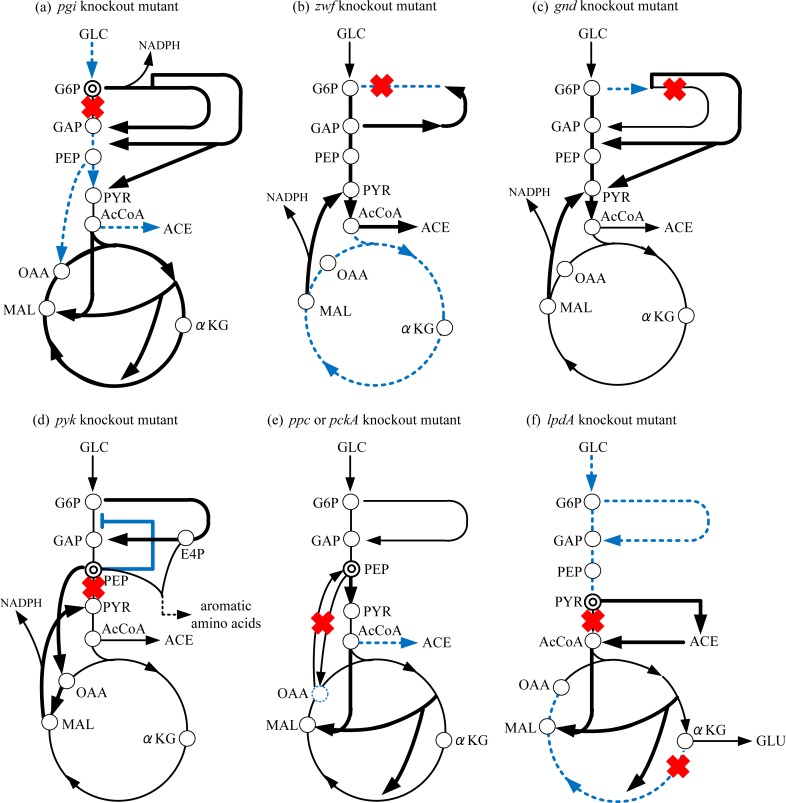
Schematic illustration for the specific gene knockout on the metabolism: (a) Δ*pgi*, (b) Δ*zwf*, (c) Δ*gnd*, (d) Δ*pyk*, (e) Δ*ppc*/pckA, (f) Δ*lpdA*.

If *pgi* gene, which codes for the first enzyme of Embden-Meyerhof-Parnas (EMP) pathway, was knocked out, the glucose catabolism occurs exclusively through the oxidative pentose phosphate (PP) pathway. As a result, NADPH is overproduced, and inhibits allosterically the activity of G6PDH, thereby reducing the glucose uptake rate, resulting in the low growth rate (Toya et al., 2010). In this mutant, the Entner-Doudoroff (ED) pathway is activated, thus reducing NADPH production at 6PGDH (Hua et al., 2003). The NADPH, that is overproduced, can be converted to NADH by transhydrogenase Udh, or the overproduced NADPH can be utilized for heterologous protein production such as PHB production etc. (Kabir and Shimizu, 2003, 2004). The growth rate can be recovered to some extent with such manipulations. Although EMP pathway can be backed up by rerouting PP pathway, its flux becomes low, resulting in low PEP concentration, and thus the anaplerotic flux through Ppc becomes low. This causes the activation of another anaplerotic pathway, glyoxylate pathway for compensating OAA concentration (Figure 3). Based on the consideration as discussed in the previous section, the decrease in the glucose uptake, or the decreased activity of PTS, causes phosphorylated EIIA (EIIA-P) to be increased, and thus cAMP-Crp increases, which in turn activates TCA cycle (Table 1). Moreover, the decreased FDP concentration (Toya et al., 2010) activates Cra activity, which in turn activates *icdA* and *aceA/B* which encode the glyoxylate pathway enzymes.

In the case of *zwf* gene knockout, the cell growth phenotype is little affected, where the non-oxidative PP pathway flux is reversed (Zhao et al., 2004a; Hua et al., 2003). This mutant shows significant overflow metabolism, and some of the NADPH, which cannot be produced at the oxidative PP pathway, is backed up by activating Mez (Figure 3), and by the transhydrogenase Pnt from NADH (Hua et al., 2003). Note that *zwf* gene knockout causes the increase in glycolysis flux, and thus EIIA-P decreases and in turn cAMP-Crp decreases. This causes TCA cycle to be repressed. Moreover, as glycolysis flux increases, FDP concentration also increases, and in turn represses Cra activity, and thus TCA cycle is repressed by the down regulation of *icdA* and *aceA/B* genes. This is the reason why more acetate overflow metabolism was observed as compared to wild type strain (Zhao et al., 2004; Hua et al., 2003).

The effect of *gnd* gene knockout on the metabolism is a little different from the case of *zwf* gene knockout, where Entner-Doudoroff (ED) pathway is activated, and the flux through G6PDH is reduced for the *gnd* mutant (Figure 3) (Zhao et al., 2004b). The reduced NADPH caused by the reduced flux through G6PDH and the disruption of 6PGDH can be backed up by activation of Mez as well as the activation of Pnt for the conversion of NADH to NADPH. Thus the cell growth phenotype is little affected by *gnd* gene knockout (Zhao et al., 2004).

In the case of *pyk* gene knockout (Δ*pykF* or Δ*pykF*, *A*), PEP is accumulated, which causes the activation of Ppc and increases the Ppc flux. This in turn causes the increased fluxes through MDH (reverse direction) and Mez, and the reduced PYR (caused by Pyk disruption) could be backed up by these alternative pathway fluxes (Figure 3) (Siddiquee et al., 2004a, b; Toya et al., 2010). Note that the accumulation of PEP, and thus EIIA-P increases, and in turn cAMP increases to some extent (Cunningham et al., 2009). However, this increase is counterbalanced by the accumulation of G6P. Namely, although the accumulation of PEP causes the activation of PTS, PEP inhibits allosterically the activation of Pfk. This in turn causes the accumulation of G6P, and then causes the degradation of mRNA of *ptsG*, while causes the activation of the oxidative PP pathway. The accumulation of PEP and the activation of PP pathway (for the production of E4P) both activate the aromatic amino acid biosynthetic pathways (Kedar et al., 2007). In fact, it has been reported that shikimic acid could be implemented by *pyk* gene knockout in the PTS^-^ background (Escalante et al., 2010). The effect of *pyk* gene knockout in the lysine producing *C. glutamicum* strain on the metabolic regulation was also investigated for strain improvement (Becker et al., 2008).

If either the *ppc* or *pckA* gene, which code for the anaplerotic and gluconeogenic pathway enzymes, respectively, was knocked out, the OAA concentration decreases, which in turn activates another anaplerotic pathway such as glyoxylate pathway (Figure 3) (Laporte and Koshland, 1982; Yang et al., 2003; Peng et al., 2004). The activation of glyoxylate pathway causes less production of acetate and CO_2_, resulting in the increase in the cell yield with lower cell growth rate as compared to wild type strain (Yang et al., 2003; Peng et al., 2004). The metabolic regulation mechanisms for these mutants seem to be similar. Namely, either *ppc* or *pckA* gene knockout causes PEP concentration to be increased, and in turn FDP and G6P concentrations increase (Yang et al., 2003; Peng et al., 2004). The increase in FDP may repress Cra activity, and in turn activates *pfkA* and *pykF* gene expressions (Table 1). Note that although Pyk activity increased, the Pfk activity decreased, which may be due to the inhibition caused by the increase in PEP concentration. Moreover, the decrease in Cra activity may decrease *icdA* and *aceA/B* gene expressions. However, the metabolic regulation at the branch point of isocitrate (ICT) is regulated by the phosphorylation /dephosphorylation of ICDH by *aceBAK*, and the decrease in OAA becomes dominant for the activation of glyoxylate pathway due to the importance of cell synthesis.

If *lpdA* gene, which codes for the subunit of PDHc, subunit of αKGDH, and glycine cleavage multi enzyme system, was knocked out, pyruvate and L-glutamate were accumulated under aerobic condition (Li et al., 2006). The metabolic flux analysis of *lpdA* gene knockout mutant indicates that glyoxylate pathway was activated, while glycolysis, the oxidative PP pathway, and the TCA cycle activities were down-regulated (Li et al., 2006a). In the case of *lpdA* gene knockout, pyruvate (PYR) accumulates and in turn glycolysis intermediates accumulate, and thus the glucose consumption rate decreases. Moreover, the accumulation of PYR activates Pox pathway producing acetate, and in turn AcCoA is backed up by ACS pathway from acetate (Figure 3) (Li et al., 2006). Since acetate once formed by Pox is assimilated through ACS, FadR and IclR decrease, and *aceBAK* operon was induced to activate glyoxylate pathway. If *sucA* gene, which codes for subunit of αKGDH, was knocked out, the metabolism is a little different from the case of *lpdA* gene knockout (Li et al., 2006b).

Under anaerobic or microaerobic conditions, NADH balance and the substrate level phosphorylation for the production of ATP may control the metabolic fluxes at the branch points such as PEP, PYR, and AcCoA (Zhu and Shimizu, 2004). As a result, *pflA*, *B* gene knockout caused overproduction of lactate (Zhu and Shimizu, 2004, 2005). In this case, PEP accumulates and in turn FDP accumulates, and repress Cra activity. This causes the activation of *pfkA* and *pykF* gene expressions, and thus the glucose uptake rate was increased (Zhu and Shimizu, 2004). If *ldhA* gene was knocked out, the fluxes toward the production of acetate, formate, and ethanol were increased as expected (Kabir et al., 2005).

The above explanation is for *E. coli*. The metabolisms of other microorganisms have also been investigated by Fuhrer et al. (2005). The above analysis is mainly made based on ^13^C-metabolic flux distributions, where the changes in the metabolic flux distributions are caused by the regulation of enzymes and the intracellular metabolite concentrations. In addition to allosteric regulation, the enzyme activities are controlled by their corresponding gene expressions. Since gene expressions are under control of global regulators or transcription factors, it is quite important to understand how these control the metabolism for metabolic engineering (Matsuoka and Shimizu, 2011). Note that Table 1 gives the relationship between transcription factor and the regulated main metabolic pathway genes, where this gives part of the whole regulation system (Gama-Castro et al., 2010)

## 5. Concluding remarks

Although most of the metabolic pathway engineering practices have focused on the modulation of the metabolic pathways of interest, the present analysis indicates the importance of understanding the overall main metabolic regulation in response the specific pathway mutation for the efficient metabolic engineering. It may be also considered to modulate transcription factors for the significant strain improvement.
